# Combination Therapy with Empagliflozin and Insulin Results in Successful Glycemic Control: A Case Report of Uncontrolled Diabetes Caused by Autoimmune Pancreatitis and Subsequent Steroid Treatment

**DOI:** 10.1155/2019/9415347

**Published:** 2019-02-14

**Authors:** Miyako Kishimoto, Kazuhide Yamaoki, Masayuki Adachi

**Affiliations:** ^1^Clinical Research Center, Department of Medicine, International University of Health and Welfare, Tochigi, Japan; ^2^Department of Internal Medicine, Sanno Hospital, 8-10-16 Akasaka, Minato, Tokyo 107-0052, Japan

## Abstract

A 66-year-old Japanese male presented with thirst, polyuria, and hemoglobin A1c and postprandial glucose levels (13.1% and 529 mg/dL, respectively) that indicated severe hyperglycemia. Based on his high immunoglobulin G4 level and the results of magnetic resonance imaging and magnetic resonance cholangiopancreatography, we diagnosed him with autoimmune pancreatitis. Insulin was initiated to control his diabetes. One month later, the patient commenced on prednisolone therapy for the treatment of autoimmune pancreatitis, after which his total insulin dosage increased to a maximum of 52 units/day. When the prednisolone dosage was later tapered, the patient's total dosage of insulin was reduced to 42 units/day. However, he had gained 3.6 kg from the start of prednisolone therapy, and 42 units/day was insufficient for maintaining glycemic control. Thus, empagliflozin, a sodium-dependent glucose transporter 2 (SGLT2) inhibitor, was added. Thereafter, we were able to reduce the patient's total dosage of insulin; it was eventually discontinued with good glycemic control and weight loss. Such results suggest that the combination of insulin with an SGLT2 inhibitor may be a viable option for the treatment of diabetic patients on prednisolone therapy.

## 1. Introduction

Sodium-dependent glucose transporter 2 (SGLT2) is a protein in the early proximal tubule that reabsorbs the majority of filtered glucose. Inhibitors of SGLT2 enhance urinary glucose excretion, thereby lowering blood glucose levels in an insulin-independent manner. SGLT2 inhibitors have pleiotropic actions, including reduced glomerular hyperfiltration, hypertension, and weight loss [1], which may correlate with reduced cardiovascular risk. In a recent study of patients with type 2 diabetes who were at high risk for cardiovascular events, those who received empagliflozin (an SGLT2 inhibitor) in addition to standard care had lower rates of the primary composite cardiovascular outcome and death from any cause than did those on placebo [2, 3]. As a result of mounting evidence, the American Diabetes Association and the European Association for the Study of Diabetes recently updated their position statements on the management of type 2 diabetes in adults [4, 5]. In their statements, an SGLT2 inhibitor with proven benefit is recommended for the treatment of patients with chronic kidney disease or clinical heart failure and atherosclerotic cardiovascular disease.

Autoimmune pancreatitis (AIP) is a chronic and progressive inflammatory pancreatic disease that is uniquely characterized by diagnostic images of pancreatic enlargement and irregularly narrowed main pancreatic ducts. It is a condition that responds dramatically to corticosteroid therapy [6–8]. Corticosteroids are frequently used for the treatment of inflammatory conditions and autoimmune diseases, but are widely recognized to cause hyperglycemia and insulin resistance when used at high doses and for long durations [9, 10].

Herein, we report the case of a patient in whom uncontrolled diabetes as a direct result of AIP and subsequent steroid treatment was successfully treated by the addition of empagliflozin to his insulin therapy.

## 2. Case Report

A 66-year-old Japanese man, 177 cm tall and weighing 66 kg (body mass index of 21.1), had been treated for hypertension for more than seven years. He had yearly medical evaluations but was never diagnosed with diabetes (postprandial glucose and hemoglobin A1c [HbA1c] levels in March 2017: 141 mg/dL and 5.4%, respectively). However, results of an annual medical check-up in March 2018 showed remarkable elevation of postprandial glucose and HbA1c levels (265 mg/dL and 11.4%, respectively). The following month (April), he reported symptoms of thirst and polyuria. His postprandial glucose and HbA1c levels on that day were 529 mg/dL and 13.1%, respectively. A high glycoalbumin level (43.2%) also suggested acute glucose elevation (Table 1). The patient's anti-glutamic acid decarboxylase antibody test was negative; however, because his postprandial C-peptide level was low (1.15 ng/mL), the patient's pancreas presumably had reduced insulin-secreting capacity. We noted that the patient's daily life had not changed in years; and he had no diabetic complications such as retinopathy, nephropathy, or neuropathy.

**Figure 1:**
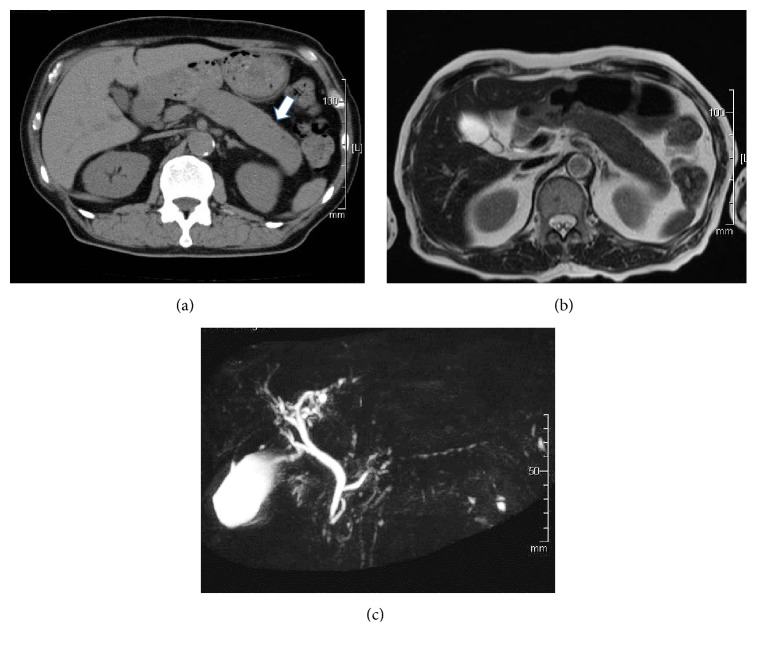
(a) An abdominal computed tomography scan performed on the patient's first visit shows diffuse swelling extending from the pancreatic body to tail. The arrow indicates the affected portion of the pancreas. (b) Magnetic resonance image (T2 weighted image) of the abdomen prior to prednisolone therapy reveals diffuse swelling extending from the pancreatic body to tail. (c) MRCP prior to prednisolone therapy shows narrowing of the main pancreatic duct extending from the pancreatic body to tail.

To identify the cause of hyperglycemia, we performed several imaging studies. Abdominal computed tomography, magnetic resonance imaging, and magnetic resonance cholangiopancreatography (MRCP) revealed diffuse swelling that extended from the pancreatic body to tail (Figures 1(a)–1(c)). In addition, MRCP showed narrowing of the associated main pancreatic duct (Figure 1(c)). The patient did not complain of any digestive symptoms such as upper abdominal pain; however, based on the imaging scans and elevation of serum immunoglobulin G4 (IgG4) levels (141.0 mg/dL), we diagnosed him with type 1 AIP.

**Figure 2:**
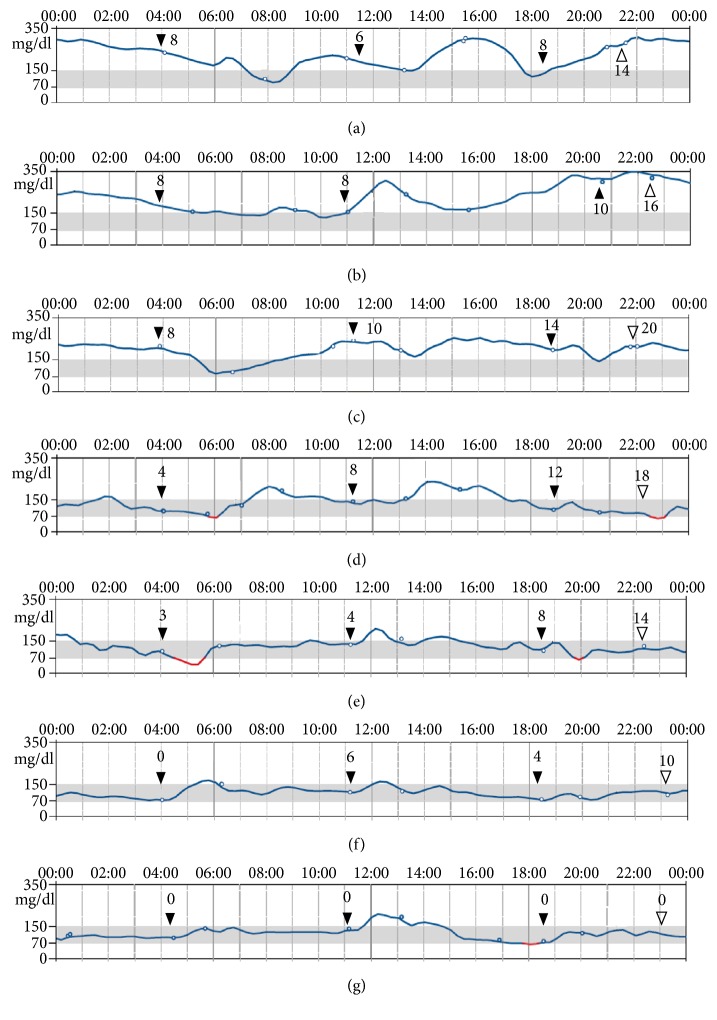
The results of continuous glucose monitoring (CGM) with the flash glucose monitoring system. Closed arrowheads indicate the timing of the patient's meals and insulin injections. Open arrowheads indicate the timing of the patient's insulin injections before sleep. Values adjacent to the arrowheads indicate the number of units of insulin injected. (a) One representative pattern of CGM prior to initiation of prednisolone. (b) Initiation of 35 mg/day of oral prednisolone. (c) Two days after initiation of prednisolone. Total dosage of insulin was increased to 52 units/day. (d) Because of the amelioration of AIP, prednisolone dosage was reduced to 25 mg/dL; however, 42 units/day of insulin was required to maintain glycemic control. (e) First day of empagliflozin administration. Hypoglycemia recorded at 5 AM to 6 AM and approximately 8 PM. (f) Twenty days after empagliflozin initiation. (g) CGM pattern of patient on empagliflozin only.

To control diabetes, the patient began self-administering insulin injections: insulin aspart (Novo Nordisk) three times per day before each meal and insulin degludec (Novo Nordisk) before going to bed. Because tight adjustment of insulin dosage is required for achieving good glycemic control, the patient received a flash glucose monitoring system (Freestyle Libre™; Abbott Diabetes Care, Witney, UK) [11] upon initiation of insulin. He initially had considerable ketosis (Table 1), but, soon after, the levels of total ketone bodies, acetoacetate, and *β*-hydroxybutyrate declined to the normal range (36 *μ*mol/L, 12 *μ*mol/L, and 24 *μ*mol/L, respectively). By the end of April, the patient's total insulin dosage was 36 units/day (Figure 2(a)). In May, prednisolone (35 mg/day) was initiated for the treatment of AIP. At that time, 42 units/day of insulin was not sufficient to control glucose elevation (Figure 2(b)); the patient required a maximum of 52 units/day (Figure 2(c)). One month later, IgG4 levels declined to 54.3 mg/dL. The dosage of prednisolone, which was being tapered by 5 mg/day every 2 weeks, was 20~25 mg/day; and the total dosage of insulin was also lower than that of the previous month. However, 42 units/day of insulin was required to maintain glycemic control (Figure 2(d)). In addition, the combination of high-dose insulin and prednisolone caused our patient to gain 3.6 kg weight from the start of prednisolone initiation.

**Figure 3:**
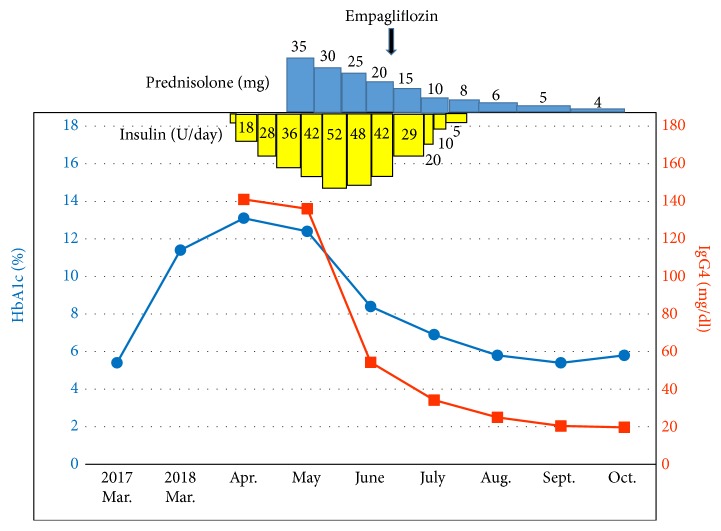
Changes in glycemic control and clinical course of AIP during prednisolone therapy. HbA1c levels (circles) and serum IgG4 levels (squares) declined over the course of treatment.

To improve glycemic control, empagliflozin was added to insulin therapy. Because we expected empagliflozin to lower blood glucose levels, we reduced the dosage of insulin to 29 units/day beforehand. Nevertheless, the patient experienced hypoglycemia 1 hour after breakfast and 1 hour after dinner on the day of empagliflozin initiation (Figure 2(e)). By the end of June, 20 days after the addition of empagliflozin, the patient had lost 1.2 kg and his total insulin dosage had declined to 20 units/day (Figure 2(f)). In July, the prednisolone dosage was reduced to 10 mg/day. Because the patient had achieved good glycemic control (postprandial glucose, HbA1c, and glycoalbumin levels: 159 mg/dL, 6.9%, and 14.3%, respectively), the total dosage of insulin was further reduced and then eventually discontinued (Figure 2(g)). Thereafter, he maintained good glycemic control (postprandial glucose and HbA1c levels: 130–180mg/dL and 5.4–5.8%, respectively) despite receiving only empagliflozin for diabetes (Figure 3). However, his postprandial C-peptide level remained low (1.84 ng/mL), revealing that although the insulin-secreting capacity of his pancreas had slightly recovered, it remained insufficient.

**Figure 4:**
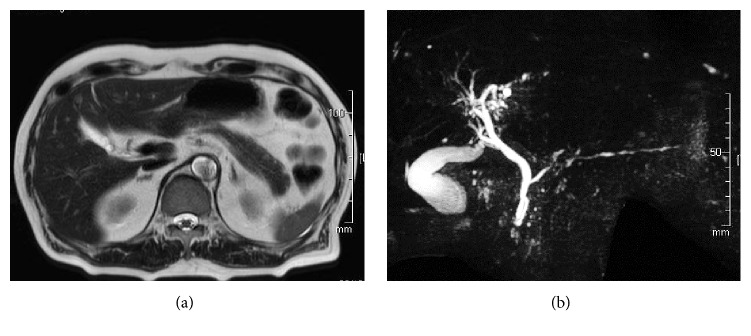
(a) Magnetic resonance image (T2 weighted image) of the abdomen after prednisolone therapy reveals amelioration of the diffuse swelling that had affected the pancreas from body to tail. (b) MRCP after prednisolone therapy revealed amelioration of the narrowing of the main pancreatic duct.

In October, the patient's prednisolone dosage was 4 mg/day. His follow-up magnetic resonance imaging and MRCP showed that both the diffuse swelling of the pancreatic tail and narrowing of the associated main pancreatic duct had been ameliorated (Figures 4(a) and 4(b)). To date, the patient's AIP is well controlled and has not relapsed.

## 3. Discussion

The international consensus diagnostic criteria for AIP identify two subtypes: type 1 is characterized by serum IgG4 elevation and the classic histopathological patterns of lymphoplasmacytic sclerosing pancreatitis; type 2 is characterized by idiopathic duct-centric pancreatitis and is not associated with IgG4 levels [12–14]. Although the clinical findings in AIP are nonspecific, the most common presentation is obstructive jaundice and upper abdominal pain [6, 7]. Approximately 40–80% of patients with AIP reportedly also present with diabetes—some with simultaneous onset with AIP and some with exacerbation of preexisting diabetes [8, 14–21]. In addition, some patients develop diabetes after the start of steroid therapy, the outcome of which varies with regard to glycemic control [17, 18, 20, 22–24].

The diabetes associated with AIP is assumed to be caused by a reduction in insulin secretion. This may be the result of various mechanisms. For one, AIP is characterized by infiltration of cluster of differentiation (CD)8 and CD4 T lymphocytes, which surround ductal cells and secrete cytokines to suppress and destroy *β*-islet cells of the pancreas [22, 23]. In addition, inflammation and fibrosis of exocrine glands is associated with the obstruction of blood flow in the endocrine glands, that results in ischemia of islet cells and dysfunctional insulin secretion. Furthermore, extensive destruction of the pancreatic islets caused by direct infiltration of inflammatory cells and fibroblasts proliferation also leads to dysfunctional insulin secretion [14, 16, 20, 23–27].

Glucocorticoids are counterhormones against insulin [9, 10], with mechanisms that include reduction of glucose uptake, induction of hepatic glucose production, and direct inhibition of insulin release [28–30]. Glucocorticoid-induced hyperglycemia is a common condition, which is often considered postprandial hyperglycemia [31, 32]. Prominent hyperglycemia is most often observed when individuals with known diabetes take high doses of glucocorticoids, but may also occur with intake of moderate and low doses in individuals without a known risk [29, 31, 33]. The odds ratio for new-onset diabetes mellitus in patients treated with glucocorticoids ranges from approximately 1.5 to 2.5, and the total glucocorticoid dose and duration of therapy are strong predictors of diabetes induction [32].

Thus, initiation of steroid therapy in patients with AIP may induce the onset of diabetes or worsen glycemic control in those with preexisting diabetes [34]. However, steroid therapy also inactivates inflammatory cells and fibroblast function, and may improve insulin secretion by controlling a series of autoimmune mechanisms, including cytokine production [16, 25]. It has been reported that pancreatic endocrine function improves after treatment with steroids in 25–45% of patients with AIP [20, 24, 35], with some patients eventually achieving a medication-free status [34]. Indeed, Miyamoto et al. reported that three months after the start of steroid therapy in patients with AIP and simultaneous onset of diabetes mellitus, 54% of patients experience improvement in diabetes, 36% do not have any change, and 9% have worsening of diabetes [16]. In addition, they noted that the long-term positive effect of corticosteroid therapy on glucose tolerance might be greater than its short-term negative effect on insulin [16].

Approximately half of patients with AIP-associated pancreatic diabetes are treated with insulin [35]. Such therapy is effective in lowering blood glucose. However, patients must be monitored for hypoglycemia and undesirable weight gain, especially when insulin dosage is increased. SGLT2 inhibitors may have glycemic benefits in patients with type 1 or type 2 diabetes who are on insulin therapy [36]. Accumulating reports indicate that SGLT2 inhibitors are well tolerated, and their addition to insulin therapy improves glycemic control and reduces body weight such that insulin dosages could sometimes be reduced [37–52]. The mechanism was explained by Ferrannini et al., who reported that empagliflozin lowers fasting and postprandial glycemia by inducing glycosuria, which improves *β* cell function and insulin sensitivity in patients with type 2 diabetes [53]. This was noted despite the fall in insulin secretion and tissue glucose disposal, and the rise in endogenous glucose production that occurs after a single dose of 25 mg empagliflozin [53]. We were able to reduce our patient's total insulin dosage upon initiation of empagliflozin. This lowered his risk of hypoglycemia and suppressed weight gain. Although improvement in glycemic control and reduction in total insulin dosage could have been the result of AIP amelioration alone, we believe that empagliflozin accelerated improvement of glycemic control in our patient.

One of the risks of SGLT2 inhibitors is diabetic ketoacidosis (DKA) [4, 5]. Similar to classic DKA, ketone accumulation in SGLT2 inhibitor-associated DKA is downstream of insulin deficiency and glucagon elevation, promoting lipolysis and hepatic ketogenesis. SGLT2 inhibitor-enhanced glucosuria effectively lowers plasma glucose levels, which decreases insulin secretion from pancreatic *β* cells. SGLT2 inhibitor-mediated glucosuria and attenuation of sodium reabsorption in the kidneys may also indirectly expand the ketone reservoir by enhancing renal ketone reabsorption [54–56]. Although our patient initially had prominent ketosis, the ketosis improved soon after initiation of insulin therapy. Although we rechecked for ketosis throughout his treatment with empagliflozin, no further signs of ketosis were detected.

In conclusion, this case report suggests that the combination of insulin and SGLT2 inhibitor can be effective for the treatment of secondary diabetes, such as prednisolone-induced diabetes associated with the treatment of AIP in our patient. Further studies on a larger scale are required to confirm the effectiveness of this combination for the treatment of diabetes in patients on corticosteroid therapy.

## Figures and Tables

**Table 1:** Postprandial laboratory results on patient's first visit.

**Hematology**		**Auto-antibody tests**	
White blood cells	7400/*μ*L	Antinuclear antibodies	< 40
Red blood cells	473×10^4^/*μ*L		
Hemoglobin	15.9 g/dL	**Immunoglobulin**	
Hematocrit	44.9%	Immunoglobulin A	186 mg/dL
Platelets	22.5×10^4^/*μ*L	Immunoglobulin M	90 mg/dL
		Immunoglobulin G	1104 mg/dL
**Blood chemistry**		Immunoglobulin G4	141 mg/dL
Albumin	4.9 g/dL		
T-bilirubin	0.8 mg/dL	**Tumor markers**	
Aspartate aminotransferase	18 IU/L	CEA	5.7 ng/mL
Alanine aminotransferase	20 IU/L	CA19-9	1.2 U/mL
Lactate dehydrogenase	162 IU/L	Span-1	< 1.0 U/mL
Alkaline phosphatase	448 IU/L	DUPAN-2	57 U/mL
*γ*-Glutamyl transpeptidase	47 IU/L		
Pancreatic amylase	13 IU/L	**Glycometabolism tests**	
Lipase	18 IU/L	Plasma glucose	529 mg/dL
Trypsin	111 ng/mL	HbA1c	13.1%
Elastase-1	93 IU/L	Glycoalbumin	43.2%
Cholinesterase	290 IU/L	C-peptide reactivity	1.15 ng/mL
Creatinine kinase	115 IU/L	Anti-GAD antibodies	< 5.0 U/mL
Uric acid	4.3 mg/dL		
Blood urea nitrogen	11.5 mg/dL	**Ketone body fractions**	
Creatinine	0.64 mg/dL	Total ketone bodies	895 *μ*mol/L
eGFR	95 ml/min/1.73m^2^	Acetoacetate	218 *μ*mol/L
Sodium	135 mEq/L	*β*-Hydroxybutyrate	677 *μ*mol/L
Potassium	4.1 mEq/L		
Chloride	98 mEq/L	**Urinalysis**	
Triglycerides	195 mg/dL	Protein	( – )
HDL cholesterol	65 mg/dL	Glucose	4+
LDL cholesterol	115 mg/dL	Occult blood	( – )
C-reactive protein	0.13 mg/dL	Ketones	(+/–)
